# Paraneoplastic Neurologic Syndromes Associated With Merkel Cell Carcinoma

**DOI:** 10.1212/NXI.0000000000200260

**Published:** 2024-10-07

**Authors:** Nicolás Lundahl Ciano-Petersen, Sergio Muñiz-Castrillo, Macarena Villagrán-García, Antonio Farina, Alberto Vogrig, Valentin Wucher, Le Duy, Cristina Birzu, David Goncalves, Olivier Flabeau, Coline Duwicquet, Adrien Benard, Fabien Nicole, Veronique Rogemond, Geraldine Picard, Bastien Joubert, Jerome Honnorat

**Affiliations:** From the French Reference Center on Paraneoplastic Neurological Syndromes and Autoimmune Encephalitis (N.L.C.-P., S.M.-C., M.V.-G., A.F., V.W., L.D.D., V.R., G.P., B.J., J.H.), Hospices Civils de Lyon, Hôpital Neurologique, Bron; MeLiS - UCBL-CNRS UMR 5284 - INSERM U1314 (N.L.C.-P., S.M.-C., M.V.-G., A.F., V.W., L.D.D., V.R., G.P., B.J., J.H.), Université Claude Bernard Lyon 1, France; Instituto de Investigación Biomédica de Málaga y Plataforma en Nanomedicina-IBIMA Plataforma BIONAND (N.L.C.-P.); Red Andaluza de Investigación Clínica y Traslacional en Neurología (NeuroRECA) (N.L.C.-P.), Málaga, Spain; Center for Sleep Sciences and Medicine (S.M.-C.), Stanford University, Palo Alto, CA; Department of Neuroscience (A.F.), Psychology, Pharmacology and Child Health. University of Florence, Italy; Clinical Neurology (A.V.), Santa Maria della Misericordia University Hospital, Azienda Sanitaria Universitaria Friuli Centrale (ASU FC); Department of Medicine (DMED) (A.V.), University of Udine, Udine, Italy; Sorbonne Université (C.B.), Inserm, CNRS, UMR S 1127, Institut du Cerveau, ICM, AP-HP, Hôpitaux Universitaires La Pitié Salpêtrière - Charles Foix, Service de Neurologie 2-Mazarin; OncoNeuroTox Group (C.B.), Center for Patients with Neurological Complications of Oncologic Treatments, GH Pitié-Salpetrière et Hôpital Percy, Paris; Immunology Department (D.G., F.N.), Hôpital Lyon Sud, Hospices Civils de Lyon, Pierre-Bénite; Service de Neurologie (O.F.), Centre Hospitalier de la Côte Basque, Bayonne; Department of Neurology (C.D.), University Hospital of Tours; and Service de Neurologie (A.B.), Centre Hospitalo-Universitaire Rennes, France.

## Abstract

**Background and Objectives:**

To define the clinical and immunologic profile of patients with paraneoplastic neurologic syndromes (PNSs) associated with Merkel cell carcinoma (MCC).

**Methods:**

Retrospective analysis was conducted on patients with suspected MCC-related PNS assessed at the French Reference Center, and cases were identified by a systematic review of the literature (MEDLINE, Embase) following the Preferred Reporting Items for Systematic Reviews and Meta-Analyses guidelines.

**Results:**

A total of 17 patients were identified in our center and 30 in the systematic review, resulting in an overall cohort of 47 patients. The median age was 65 years (range 41–90), and 30 of 46 (65%) were men. Lambert-Eaton myasthenic syndrome (LEMS) (14/47, 29%), rapidly progressive cerebellar syndrome (11/47, 23%), and encephalomyelitis (EM) (8/47, 17%) were the most common associated clinical phenotypes. The most frequently associated neural antibodies (Abs) were voltage-gated calcium channel (VGCC)-Abs (14/45, 31%), followed by Hu-Abs (8/45, 17%) and neurofilament (NF)-Abs (8/45, 17%). Patients with NF-Abs only exhibited CNS disorders (8/8, 100%) and often had antibodies against >1 NF subunit (6/8, 75%). At onset, 26 of 43 patients (60%) had no identifiable primary skin tumor but had lymph node metastasis; these patients were more frequently men (21/26, 80%, vs 7/17, 41%; *p* = 0.007), had more frequently VGCC-Abs (12/26, 46%, vs 2/17, 11%, *p* = 0.02) predominantly among those with LEMS, and presented reduced mortality than patients with a known primary tumor (5/25, 20%, vs 8/15, 53%; *p* = 0.02).

**Discussion:**

MCC-related PNSs present as a heterogeneous clinical spectrum including central and/or peripheral nervous system disorders such as LEMS, RCPS, and EM, mainly associated with VGCC-Abs, NF-Abs, and Hu-Abs. NF-Abs were only seen among patients with CNS disorders. At onset, the absence of a primary skin tumor but presence of lymph node metastasis is frequently observed, and this particular clinical presentation is linked to reduced mortality, highlighting distinctive clinical and immunologic features of MCC-related PNS.

## Introduction

Paraneoplastic neurologic syndromes (PNSs) are immune-mediated disorders triggered by an underlying systemic malignancy. The most frequently associated tumors are lung, breast, and gynecologic cancers, as well as lymphomas.^1-3^ However, the causal association with other tumors such as Merkel cell carcinoma (MCC) remains poorly investigated despite neuroendocrine cancers being widely associated with PNS.^4^

MCCs are rare neuroendocrine skin tumors that predominantly affect older White men. They are considered the most lethal skin malignancy because of their ability to rapidly spread to regional lymph nodes and distant organs.^5^ Heavy exposure to ultraviolet (UV) light and advanced age have classically been defined as the main risk factors of MCC.^6^ In addition, its high incidence among chronically immunosuppressed patients led to the identification of Merkel cell polyomavirus (MCPyV) as a major oncogenic factor in up to 80% of MCCs, suggesting a key role of the immune system in its development.^7^ Furthermore, MCCs are highly immunogenic tumors due to the frequent expression of MCPyV oncoproteins and their high somatic mutation burden leading to the generation of neoepitopes.^6,8^ These intrinsic MCC features may enhance the antitumor immune response, probably explaining their good response to immune checkpoint inhibitors,^9^ but also their ability to break immune-tolerance mechanisms leading to PNS.

In this article, we describe the clinical presentation and associated antibody (Ab) profiles of patients with suspected PNS in association with MCC diagnosed at the French Reference Center for Paraneoplastic Neurological Syndromes and Autoimmune Encephalitis. In addition, we retrieved previously reported cases by performing a systematic review of the literature.

## Methods

### Patient Selection From the French Reference Center

For the purpose of this study, we retrospectively screened the database of the French Reference Center for Paraneoplastic Neurological Syndromes and Autoimmune Encephalitis (Lyon, France) for patients with suspected PNS associated with MCC between January 1, 2007, and June 1, 2023. The inclusion criteria were the development of neurologic symptoms within 2 years from MCC diagnosis, absence of another more frequently associated cancer with the PNS, and absence of a better alternative diagnosis. Only patients who met all the inclusion criteria were selected.

NLC-P performed the initial search in the electronic database and the collection of data from the medical charts. If relevant information was not available, the referring physicians of the patients were contacted. The final decision to include the patient was reached after a consensus-based discussion between NLC-P and JH. Patients included in the study were classified according to the predominant neurologic syndrome as previously described.^4,10^ Ancillary test findings including CSF analysis, MRI, and neurophysiologic studies were also extracted. MRI was considered as abnormal in the presence of parenchymal T2 hyperintensities, meningeal enhancement, or cerebellar atrophy. Inflammatory CSF was defined by pleocytosis ≥5 cells/mL, presence of oligoclonal bands, and/or CSF protein level >0.45 mg/dL. EEG was considered as abnormal in the presence of epileptic discharges and/or focal/generalized slowing. Abnormal electroneurogram-electromyogram (ENG-EMG) was defined by the presence of myopathic findings, axonal or mixed neuropathy, sensory neuronopathy (SNN), and/or neuromuscular junction dysfunction. The PNS-Care Score was calculated according to the updated diagnostic criteria for PNS.^4^ Regarding the cancer association, MCC scored 4 points because of its neuroendocrine nature, except for patients with a low-risk phenotype regardless of the Ab status.^4^ Regarding tumor staging, spontaneous primary tumor regression was suspected when lymph node metastasis was observed through lymph node biopsy, without an identifiable primary skin tumor after a dermatological evaluation at any point during the course of the disease. Clinical outcomes were classified into 4 categories according to the clinical description on follow-up records: recovery, partial recovery, no improvement or disease progression, or death.

### Systematic Review

The review protocol followed the declaration of Preferred Reporting Items for Systematic Reviews and Meta-Analyses (PRISMA) (Figure). A systematic search of articles published from April 1, 1995, to August 1, 2022, was performed on MEDLINE/PubMed and Embase databases. The following MeSH terms, free text, and related search terms were included: “Merkel cell carcinoma” AND (“paraneoplastic” OR “neurology” OR “neurologic complication” OR “autoimmunity” OR “autoimmune disease” OR “autoantibodies”). The first screening of results by titles and abstracts was performed, and only studies with full text available in English or French language were included. Additional articles were retrieved by searching through the reference lists of all studies. All studies were carefully evaluated to ensure that the cases extracted had not been previously included. NLC-P performed the initial selection of relevant studies and extracted the information from each article. Whenever the retrieved information was unclear or there was uncertainty about the inclusion of one report or patient, the decision was taken after a consensus-based discussion. JH supervised the entire systematic review process.

**Figure:**
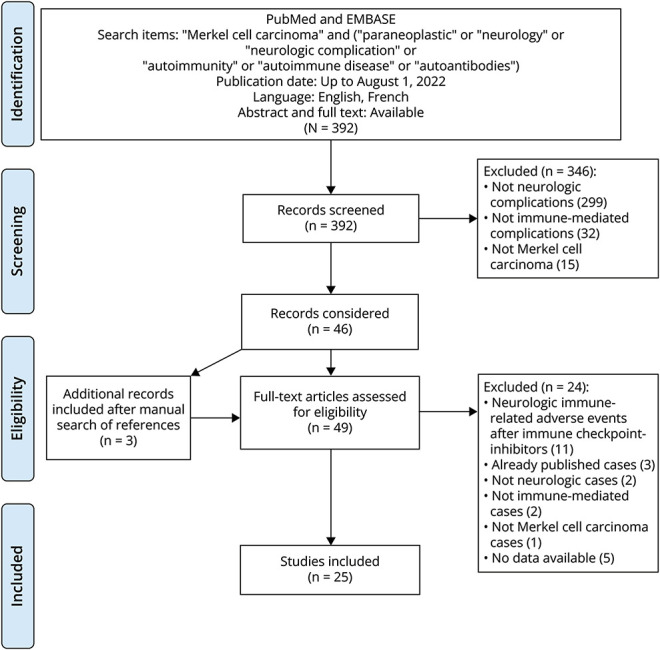
Flow Diagram Representing the Main Phases of the Systematic Review Detailing the Number of Studies Screened, Included, and Excluded

The same inclusion criteria for the selection of the patients from the French Reference Center were applied in the systematic review. Whenever available, results of paraclinical studies, treatment, and outcomes were also registered. Patients included in the systematic review and in the cohort of the French Reference Center were pooled together and further classified according to the predominant clinical manifestations into 5 main groups: Lambert-Eaton myasthenic syndrome (LEMS), rapidly progressive cerebellar syndrome (RPCS), encephalomyelitis (EM), other CNS disorders, and other neuromuscular (NM) disorders. Clinical outcomes were categorized into 4 categories as stated in the previous section.

### Autoantibody Identification

For the systematic neural Ab screening, serum and/or CSF samples of patients were tested by indirect immunofluorescence on rat brain sections, further confirmed by a cell-based assay (CBA) using HEK293 cells expressing the suspected target antigen, as previously described.^11^ Diagnosis was only established when both techniques were positive. All patients with an indirect immunofluorescence staining pattern compatible with neurofilament (NF)-Abs were further tested with CBA transiently transfected to express heavy-chain NF, medium-chain NF, light-chain NF, or α-internexin, following the approach of the original report.^12^ By contrast, voltage-gated calcium channel (VGCC)-Abs were identified by radioimmunoassay or ELISA at the French reference center for the detection of these autoantibodies (Marseille, France).

### Statistical Analysis

Continuous variables are described by median and range while qualitative variables are described by absolute and relative frequencies for each category of the variable (missing data not included). Comparisons between groups were performed using the Wilcoxon-Mann-Whitney test for continuous variables and the χ^2^ test or the Fischer exact test for qualitative variables. Statistical analyses were performed using software IBM SPSS Statistics, version 29.0. All *p* values were two-tailed, and *p* values < 0.05 were considered statistically significant.

### Standard Protocol Approvals, Registrations, and Patient Consents

The Institutional Review Board of the University Claude Bernard Lyon 1 and Hospices Civils de Lyon approved the study (NCT03963700), which was performed in accordance with the Code of Ethics of the World Medical Association (Declaration of Helsinki). Written consent was obtained from all patients for the storage and use of their clinical information and biological samples for research purposes.

### Data Availability

Anonymized data not published within this article will be made available by request from any qualified investigator.

## Results

### French Reference Center Cohort

A total of 17 patients with PNS associated with MCC diagnosed between 2007 and 2023 were identified in the French Reference Center; the median age was 70 years (range 41–88), and 9 of 17 (52%) were men. Among them, 4 of 17 (23%) had EM, 4 of 17 (23%) had RPCS, 3 of 17 (17%) had LEMS associated with cerebellar dysfunction, 2 of 17 (11%) had brainstem encephalitis, and 4 of 17 (23%) had other isolated syndromes (1 autonomic neuronopathy, 1 extralimbic encephalitis, 1 isolated myelitis, 1 motor neuron disease; Table 1). Patient 17 was surprising because of the clinical presentation with motor neuron dysfunctions that are unusual in PNS, but he had VGCC-Abs and we were unable to definitively exclude a LEMS. Neural Abs were found in 16 of 17 patients (94%); among them, 4 of 17 (23%) had high-risk Abs (2 CV2/CRMP5-Abs, 2 Hu-Abs) and 3 of 17 (17%) had intermediate-risk Abs such as VGCC-Abs.^4^ In addition, we observed the presence of atypical Abs identified by tissue-based indirect immunofluorescence in 10 of 17 patients (58%): 2 (11%) had Abs against unknown neural antigens, and 8 of 17 (47%) had a NF-like staining by immunofluorescence. Among them, 6 of 8 (75%) recognized different NF subunits using CBAs while 2 of 8 (25%) did not recognize any of the previously described NF.^12^

**Table 1:** French Cohort of Patients With MCC-Related PNS

Nº/Age	Clinical picture	First diagnosis–delay^a^	Tumor status at PNS diagnosis	Antibody	PNS-Care Score	MRI	CSF	ImmunoT	OncoT	Comments
1/7th decade	LEMS	PNS–2 mo	Lymph node, primary unknown	CSF VGCC + atypical anti-NF^b^	9	Normal	11 WBCs	Steroids, IVIG, RTX, CPP	Surgery, RT, CT	Tumor MCPyV+ RPCS associated
2/6th decade	LEMS	Tumor–6 mo	Lymph node	Negative	7	Normal	OCB	Steroids, IVIG, PLEX, RTX, MM	Surgery, RT	RPCS associated
3/6th decade	LEMS	PNS–2 mo	Lymph node	CSF unknown neuropil Ab	7	Leptomeningeal enhancement	Prot 74 mg/dL	Steroids, IVIG, CPP	Surgery	Tumor MCPyV+ RPCS associated
4/9th decade	RPCS	PNS–1 mo	NA	CSF atypical anti-NF^b^	7	Normal	Prot 80 mg/dL	No	No	
5/5th decade	RPCS	PNS–1 mo	Lymph node, primary unknown	CSF VGCC Anti-NF medium chain	9	T2 HS in the cervical spinal cord, cerebellum, and conus medullaris	9 WBCs, Prot 80 mg/dL	Steroids, IVIG, CPP	Surgery, RT	Associated myelitis
6/6th decade	RPCS	PNS–2 mo	Local tumor	CSF unknown antineuronal Ab	7	Normal	NA	No	Surgery, RT, CT	
7/8th decade	RPCS	PNS–1 mo	Lymph node, primary unknown	CSF anti-NF heavy, medium, and light chains	7	Normal	7 WBCs, Prot 66 mg/dL	IVIG	No	
8/8th decade	EM	PNS–7 mo	Lymph node	Serum GAD >1/4,000	7	Normal	55 WBCs	No	Surgery, RT	
9/8th decade	EM	PNS–8 mo	Lymph node, primary unknown	Serum CV2/CRMP5	10	T2 HS in bilateral caudate and lenticular nucleus	Prot 245 mg/dL	Steroids, IVIG, CPP	CT, RT	
10/8th decade	EM	Tumor–12 mo	Lymph node	Serum Hu	10	T2 white matter HS	Normal	Steroids	Surgery, RT	
11/8th decade	EM	PNS–6 mo	Lymph node, primary unknown	CV2/CRMP5	10	Basal ganglia HS	250 mg/dL	Steroids, IGIV, CPP	Qx, RT, QT	
12/7th decade	Brainstem encephalitis	PNS–1 mo	Lymph node, primary unknown	CSF anti-NF medium chain	6	T2 HS in bilateral superior cerebellar peduncles	Normal	Steroids, IVIG, PLEX	Surgery, RT	
13/8th decade	Brainstem encephalitis	PNS–1 mo	Lymph node, primary unknown	CSF anti-NF medium, light, and alfa-internexin chains	6	Normal	NA	IGIV	No	
14/6th decade	Extralimbic encephalitis	Tumor–13 mo	Local tumor	CSF anti-NF heavy, medium, light, and alfa-internexin chains	6	Diffuse WM HS	380 WBCs, 250 mg/dL	Steroids, IGIV	Qx	Bilateral optic neuritis and Sjogren syndrome
15/8th decade	Myelitis	PNS–1 mo	Lymph node, primary unknown	CSF anti-NF heavy, medium, light, and alfa-internexin chains	6	C2-C5 + T2 HS	50 WBCs, 190 mg/dL	Steroids, PLEX	Qx, RT	
16/9th decade	Autonomic neuronopathy	Tumor–2 mo	Lymph node	Serum Hu	10	NA	Prot 66 mg/dL	No	Surgery, RT	
17/7th decade	Motor neuron disease	PNS–3 mo	Lymph node	Serum VGCC	3	Normal	NA	Steroids, cyclosporine (kidney graft)	Surgery, RT	

Abbreviations: Ab = antibody; CT = chemotherapy; EM = encephalomyelitis; GAD = glutamate decarboxylase; HS = hyperintensity; ICI = immune checkpoint inhibitor; ImmunoT = immunotherapy; IVIG = IV immunoglobulin; LEMS = Lambert-Eaton myasthenic syndrome; NA = not available; OCB = oligoclonal band; OncoT = oncotherapy; MCPyV = Merkel cell polyomavirus; ML = molecular layer; NF = neurofilament = ON = optic neuritis; PLEX = plasma exchange; PNS = paraneoplastic neurologic syndrome; RPCS = rapidly progressive cerebellar syndrome; RT = radiotherapy; VGCC = voltage-gated calcium channel; WBCs = white blood cells; WM = white matter.

a Delay was defined as the time between the diagnosis of the PNS and that of the tumor or from the diagnosis of the tumor and that of the PNS.

b These patients had a neurofilament-like staining on tissue-based immunofluorescence but were negative on cell-based assays transiently transfected to express heavy-chain NF, medium-chain NF, light-chain NF, or α-internexin.

### Systematic Review of the Literature and Total Cohort Description

A total of 392 articles were screened, and 30 patients with PNS associated with MCC were identified in 25 different studies (Figure). Clinical and paraclinical details of these patients are presented in eTables 1–5 according to the clinical phenotype. No significant differences were observed between the French Reference Center cohort and patients reported in the literature (eTable 6), except for a higher rate of Abs against unknown antigens in our series (10/17, 58%, vs 4/28, 14%, *p* = 0.002), more frequent administration of second-line immunotherapies other than rituximab (7/17, 41%, vs 0/22, 0%, *p* = 0.001), and lower rate of complete recovery (0/16, 0%, vs 8/27, 29%; *p* = 0.01), probably related to the referral of patients experiencing a higher degree of severity.

Thus, to better define MCC-related PNS, both cohorts were combined, resulting in a total of 47 patients (Table 2). The median age was 65 years (range 41–90), and 30 of 46 (65%) were men. The most common clinical phenotype was LEMS in 14 of 47 (29%), followed by RPCS in 11 of 47 (23%) and EM in 8 of 47 (17%). Of interest, 5 of 14 patients with LEMS (35%) also had cerebellar dysfunction, increasing the frequency of cerebellar involvement to 16 of 47 (34%). Neural Abs were identified in 38 of 45 patients (84%): 10 of 45 (22%) had high-risk Abs such as Hu-Abs (8/45, 17%) or CV2/CRMP5-Abs (3/45, 6%); 1 patient (2%) had both Hu-Abs and CV2/CRMP5-Abs; 15 of 45 (33%) had intermediate-risk Abs, mainly VGCC-Abs (14/45, 31%); and 2 of 45 (4%) had low-risk Abs targeting GAD65. Of interest, Abs against different NFs were found in 8 of 45 patients (17%), all with CNS disorders and, in most cases, against more than 1 NF (6/8, 75%), including heavy-chain NF (5/8, 62%), medium-chain NF (8/8, 100%), light-chain NF (6/8, 75%), α-internexin (4/8, 50%), and peripherin (1/2, 50%). Diagnostic criteria for definite PNS were met by 23 of 47 patients (48%), mostly among those with high-risk clinical phenotypes (3); by contrast, probable PNS criteria were fulfilled in 21 of 47 patients (44%), who presented either with high-risk phenotypes but without high-risk Abs or with low-risk phenotypes. Notably, all 4 patients (100%) whose tumor was analyzed for the presence of MCPyV were positive.

**Table 2:** Demographic and Clinical Features of 47 Patients Identified Through the Systematic Review Together With Those From the French Cohort

	Total cohort (n = 47)
Median age (range)	65 (41–90)
Men (n, %)	30/46 (65)
Median PNS-Care Score (range)	7 (1–10)
Tumor diagnosed before PNS (n, %)	18/46 (39)
Tumor status at PNS diagnosis (n, %)	
Local tumor	2/43 (4)
Lymph node, primary unknown	26/43 (60)
Lymph node, primary known	11/43 (25)
Distant metastasis	4/43 (9)
Paraneoplastic neurologic syndromes (n, %)	
LEMS	14/47 (29)
RPCS	11/47 (23)
EM	8/47 (17)
Other NM disorders^a^	8/47 (17)
Other CNS disorders^b^	6/47 (12)
Neural antibodies (n, %)	38/45 (84)
High-risk Abs	10/45 (22)
Intermediate-risk Abs	15/45 (33)
Low-risk Abs (n, %)	2/45 (4)
NF Abs	8/45 (17)
Immunotherapy (n, %)	
Steroids	23/39 (58)
IVIG	21/39 (53)
PLEX	4/39 (10)
Rituximab	3/39 (7)
Other immunotherapies^c^	7/39 (17)
Outcomes (n, %)	
Recovery	8/43 (18)
Partial recovery	14/43 (32)
No improvement or progression	6/43 (13)
Death	15/43 (34)

Abbreviations: Abs = antibodies; EM = encephalomyelitis; IVIG = IV immunoglobulins; NF = neurofilament; NM = neuromuscular; PLEX = plasma exchange; PNS = paraneoplastic neurologic syndrome; RPCS = rapidly progressive cerebellar syndrome.

a Other NM disorders including sensory neuronopathy (2), motor neuron disorder (1), necrotizing myopathy (1), autonomic neuronopathy (1), dermatomyositis (1), sensory-motor axonal neuropathy (1), and myositis (1).

b Other CNS disorders including brainstem encephalitis (3), limbic encephalitis (1), extralimbic encephalitis (1), and myelitis (1).

c Other immunotherapies including cyclophosphamide (5), mycophenolate mofetil (1), and cyclosporine (1).

### Patients With Suspected Spontaneous Primary Tumor Regression and MCC Lymph Node Metastasis

We then compared patients without an identifiable primary skin tumor and MCC lymph node metastasis (26/43, 60%) with those with a known primary skin tumor (17/43, 39%; eTable 7). The former were more frequently men (21/26, 80%, vs 7/17, 41%, *p* = 0.007). As expected, the diagnosis of MCC preceded the diagnosis of PNS more frequently in patients with a known primary skin tumor (13/17, 76%, vs 4/26, 15%, *p* = 0.0001). A similar distribution of clinical phenotypes was observed between both groups, with LEMS being the most frequent clinical syndrome (9/26, 34%), followed by RPCS (5/26, 19%) and EM (4/26, 15%). Accordingly, patients without an identifiable primary skin tumor and MCC lymph node metastasis had more frequently VGCC-Abs (12/26, 46%, vs 2/17, 11%, *p* = 0.02). Of interest, patients with a known primary skin tumor had a higher mortality rate (8/15, 53%, vs 5/25, 20%, *p* = 0.02).

## Discussion

The causality and possible paraneoplastic origin of autoimmune neurologic disorders in patients with MCC remain poorly defined despite the neuroendocrine nature of this rare skin malignancy. In this article, we comprehensively investigated the clinical spectrum and autoantibody profile of PNS in patients with MCC. The most common PNSs observed were LEMS, RPCS, and EM. Of interest, at onset, more than half of the patients had no identifiable primary skin tumor but presented MCC lymph node metastasis, suggesting a spontaneous regression of the primary tumor; these patients developed mainly LEMS and VGCC-Abs. Furthermore, atypical Abs against different NFs were frequently identified among patients with MCC-related PNS.

Certain malignancies are highly associated with specific PNSs such as ovarian cancer and Yo-Ab RPCS or Hodgkin lymphomas and DNER-Ab RPCS.^13,14^ Conversely, malignancies of neuroendocrine lineage such as small-cell lung cancer (SCLC) present a wider clinical spectrum, comprising the central and/or peripheral nervous system.^15^ In this study, we observed a similar heterogeneous clinical spectrum of neurologic syndromes in patients with MCC, mainly represented by LEMS, RPCS, overlapping LEMS/RPCS, and EM, in line with previous observations.^12,16,17^ However, other high-risk phenotypes frequently observed in patients with SCLC-related PNS, such as SNN or limbic encephalitis, were seldomly observed in association with MCC.

Likewise, the Ab profile of MCC-related PNS resembles that of PNS associated with SCLC because Hu-Abs, CV2/CRMP5-Abs, and VGCC-Abs were frequently detected in patients with MCC. These clinical and immunologic similarities between MCC-related and SCLC-related PNS are likely linked to their common neuroendocrine nature, as reflected by the frequent expression of the antigens targeted by the aforementioned Abs in both malignancies,^15,16,18,19^ supporting the causal association and paraneoplastic origin of MCC-related PNS.

Surprisingly, MCC-related PNSs often present other uncommon neural Abs against a broad variety of NFs. These Abs were initially identified in patients with neurodegenerative disorders, but their role as biomarkers of neurologic autoimmunity has recently been proposed.^12,20,21^ Nevertheless, only light-chain NF-Abs were strongly associated with CNS disorders, mainly RPCS, and malignancies of neuroendocrine lineage, including MCC; by contrast, the remaining NF-Abs were related to less specific clinical or cancer profiles, and, occasionally, they were linked to autoimmune disorders in the context of infections such as HIV and ehrlichiosis.^12,20^ In this article, all patients with NF-Abs presented CNS disorders; however, a relation between a specific NF-Ab and a particular clinical syndrome was not observed because most patients presented Abs directed against multiple subtypes of NF, in agreement with previous studies.^12,20^ Therefore, our findings suggest that NF-Abs could be a potential biomarker of PNS associated with neuroendocrine malignancies, although future studies are required to confirm their role. Of interest, heavy-chain NF, medium-chain NF, light-chain NF, and α-internexin have been found to be expressed by the tumor of patients with MCC-related PNS, supporting their potential as a biomarker of PNS.^12^ However, their sole expression by the tumors is not sufficient to develop a PNS because 75% of MCCs express NF while only a minority develop a PNS,^22-24^ similar to what is observed in patients with SCLC.^25^ Therefore, despite an ectopic antigen expression seem to be one of the pathogenic mechanisms, other yet unidentified factors likely play a role in the immune tolerance breakdown that defines MCC-related PNS.^26^

The involvement of regional lymph node metastasis seems to be required to trigger the immune cascade leading to MCC-related PNS. Indeed, while 70% of regular MCCs are restricted to the skin at diagnosis,^9^ at the time of PNS onset, almost all patients had macroscopic lymph node or distant metastasis. Strikingly, more than half of patients presented MCC lymph node metastasis and a suspicion of spontaneous regression of the primary skin tumor, whereas this presentation is only observed in 3% of patients with regular MCC.^27^ Moreover, patients with this particular MCC presentation were associated with reduced mortality compared with patients with an identifiable skin tumor despite having the same PNS. This phenomenon has also been described in patients with Kelch-like protein 11 encephalitis, who frequently present a burned-out testicular tumor with lymph node metastasis,^28^ and in some patients with SCLC-related PNS and Hu-Abs, who may have a spontaneous regression of the lung tumor.^29^ An enhanced antitumor immune response has been proposed to drive this primary tumor regression, supported by the complete absence of this MCC presentation among immunosuppressed patients.^8^ Of interest, this circumstance predominates among patients with VGCC-Abs and LEMS, raising the possibility of a relationship between the nature of the antitumor immune response, the targeted neuronal antigen, and the clinical phenotype. In this line, patients with a spontaneous regressed melanoma with lymph node metastasis have a two-fold higher risk of developing vitiligo than patients with known primary melanoma, suggesting an excessive antimelanocytic immune response in this scenario.^30^ A similar antineuronal immune response may drive PNS in patients with a spontaneously regressed primary MCC. However, because MCC is not considered to originate directly form nervous tissue,^9^ other immune mechanisms are likely involved in the pathogenesis of MCC-related PNS.

The presence of MCPyV in 80% of MCCs is considered as another critical factor contributing to the high immunogenicity of these tumors.^6-8^ Unfortunately, we only found 4 patients for whom the MCPyV tumor analysis was reported; therefore, we were unable to determine its role in MCC-related PNS.

Our study has several limitations intrinsic to its retrospective nature. Excluding patients with incomplete clinical records, as well as the rarity of these disorders, led to a small sample size and a limited statistical power. Furthermore, the heterogeneity in the methodology of previously reported cases may have led to biased conclusions. Future well-designed prospective studies are, therefore, required.

In conclusion, the clinical and antibody spectrum of MCC-related PNS is heterogeneous and resembles that of SCLC-related PNS. Central and/or peripheral nervous system symptoms were observed in association with VGCC-Abs, NF-Abs, Hu-Abs, and CV2/CRMP5-Abs. An efficient antitumor response seems to underlie the pathogenesis of MCC-related PNS, as reflected by the frequent absence of an identifiable primary skin tumor despite having lymph node metastasis. Future studies, however, should further investigate the precise immunologic mechanisms and assess the role of MCPyV.

## Appendix Authors

**Table:**

Name	Location	Contribution
Nicolás Lundahl Ciano-Petersen, MD	French Reference Center on Paraneoplastic Neurological Syndromes and Autoimmune Encephalitis, Hospices Civils de Lyon, Hôpital Neurologique, Bron; MeLiS - UCBL-CNRS UMR 5284 - INSERM U1314, Université Claude Bernard Lyon 1, France; Instituto de Investigación Biomédica de Málaga y Plataforma en Nanomedicina-IBIMA Plataforma BIONAND; Red Andaluza de Investigación Clínica y Traslacional en Neurología (NeuroRECA), Málaga, Spain	Drafting/revision of the manuscript for content, including medical writing for content; major role in the acquisition of data; study concept or design; analysis or interpretation of data
Sergio Muñiz-Castrillo, MD, PhD	French Reference Center on Paraneoplastic Neurological Syndromes and Autoimmune Encephalitis, Hospices Civils de Lyon, Hôpital Neurologique, Bron; MeLiS - UCBL-CNRS UMR 5284 - INSERM U1314, Université Claude Bernard Lyon 1, France; Center for Sleep Sciences and Medicine, Stanford University, Palo Alto, CA	Drafting/revision of the manuscript for content, including medical writing for content; analysis or interpretation of data
Macarena Villagrán-García, MD	French Reference Center on Paraneoplastic Neurological Syndromes and Autoimmune Encephalitis, Hospices Civils de Lyon, Hôpital Neurologique, Bron; MeLiS - UCBL-CNRS UMR 5284 - INSERM U1314, Université Claude Bernard Lyon 1, France	Drafting/revision of the manuscript for content, including medical writing for content
Antonio Farina, MD	French Reference Center on Paraneoplastic Neurological Syndromes and Autoimmune Encephalitis, Hospices Civils de Lyon, Hôpital Neurologique, Bron; MeLiS - UCBL-CNRS UMR 5284 - INSERM U1314, Université Claude Bernard Lyon 1, France; Department of Neuroscience, Psychology, Pharmacology and Child Health. University of Florence, Italy	Drafting/revision of the manuscript for content, including medical writing for content
Alberto Vogrig, MD, PhD	Clinical Neurology, Santa Maria della Misericordia University Hospital, Azienda Sanitaria Universitaria Friuli Centrale (ASU FC); Department of Medicine (DAME), University of Udine, Udine, Italy	Drafting/revision of the manuscript for content, including medical writing for content
Valentin Wucher, PhD	French Reference Center on Paraneoplastic Neurological Syndromes and Autoimmune Encephalitis, Hospices Civils de Lyon, Hôpital Neurologique, Bron; MeLiS - UCBL-CNRS UMR 5284 - INSERM U1314, Université Claude Bernard Lyon 1, France	Analysis or interpretation of data
Le Duy, Do	French Reference Center on Paraneoplastic Neurological Syndromes and Autoimmune Encephalitis, Hospices Civils de Lyon, Hôpital Neurologique, Bron; MeLiS - UCBL-CNRS UMR 5284 - INSERM U1314, Université Claude Bernard Lyon 1, France	Major role in the acquisition of data
Cristina Birzu, MD	Sorbonne Université, Inserm, CNRS, UMR S 1127, Institut du Cerveau, ICM, AP-HP, Hôpitaux Universitaires La Pitié Salpêtrière - Charles Foix, Service de Neurologie 2-Mazarin; OncoNeuroTox Group, Center for Patients with Neurological Complications of Oncologic Treatments, GH Pitié-Salpetrière et Hôpital Percy, Paris, France	Drafting/revision of the manuscript for content, including medical writing for content
David Goncalves, PharmD	Immunology Department, Hôpital Lyon Sud, Hospices Civils de Lyon, Pierre-Bénite, France	Major role in the acquisition of data
Olivier Flabeau, MD	Service de Neurologie, Centre Hospitalier de la Côte Basque, Bayonne, France	Drafting/revision of the manuscript for content, including medical writing for content
Coline Duwicquet, MD	Department of Neurology, University Hospital of Tours, France	Drafting/revision of the manuscript for content, including medical writing for content
Adrien Benard	Service de Neurologie, Centre Hospitalo-Universitaire Rennes, France	Drafting/revision of the manuscript for content, including medical writing for content
Fabien Nicole	Immunology Department, Hôpital Lyon Sud, Hospices Civils de Lyon, Pierre-Bénite, France	Major role in the acquisition of data
Veronique Rogemond	French Reference Center on Paraneoplastic Neurological Syndromes and Autoimmune Encephalitis, Hospices Civils de Lyon, Hôpital Neurologique, Bron; MeLiS - UCBL-CNRS UMR 5284 - INSERM U1314, Université Claude Bernard Lyon 1, France	Major role in the acquisition of data
Geraldine Picard	French Reference Center on Paraneoplastic Neurological Syndromes and Autoimmune Encephalitis, Hospices Civils de Lyon, Hôpital Neurologique, Bron; MeLiS - UCBL-CNRS UMR 5284 - INSERM U1314, Université Claude Bernard Lyon 1, France	Major role in the acquisition of data
Bastien Joubert, MD, PhD	French Reference Center on Paraneoplastic Neurological Syndromes and Autoimmune Encephalitis, Hospices Civils de Lyon, Hôpital Neurologique, Bron; MeLiS - UCBL-CNRS UMR 5284 - INSERM U1314, Université Claude Bernard Lyon 1, France	Drafting/revision of the manuscript for content, including medical writing for content
Jerome Honnorat, MD, PhD	French Reference Center on Paraneoplastic Neurological Syndromes and Autoimmune Encephalitis, Hospices Civils de Lyon, Hôpital Neurologique, Bron; MeLiS - UCBL-CNRS UMR 5284 - INSERM U1314, Université Claude Bernard Lyon 1, France	Drafting/revision of the manuscript for content, including medical writing for content; major role in the acquisition of data; study concept or design; analysis or interpretation of data

## Glossary

Ab: antibody
CBA: cell-based assay
EM: encephalomyelitis
LEMS: Lambert-Eaton myasthenic syndrome
MCC: Merkel cell carcinoma
NF: neurofilament
PNSs: paraneoplastic neurologic syndromes
RPCS: rapidly progressive cerebellar syndrome
SCLC: small-cell lung cancer
SNN: sensory neuronopathy
VGCC: voltage-gated calcium channel
